# Aortic Valvular Disease in Elderly Subjects with Heterozygous Familial Hypercholesterolemia: Impact of Lipid-Lowering Therapy

**DOI:** 10.3390/jcm8122209

**Published:** 2019-12-14

**Authors:** Victoria Marco-Benedí, Martin Laclaustra, Juan M. Casado-Dominguez, Rosa Villa-Pobo, Rocío Mateo-Gallego, Rosa M. Sánchez-Hernández, Marta Blanco Nuez, Emilio Ortega-Martínez de Victoria, Marta Sitges, Juan Pedro-Botet, Jose Puzo, Teresa Villarroel, Fernando Civeira

**Affiliations:** 1 Lipid Unit, Hospital Universitario Miguel Servet, IIS Aragón, CIBERCV, 50009 Zaragoza, Spain; vmarcob@iisaragon.es (V.M.-B.); rosavillapobo@hotmail.com (R.V.-P.); rmateo@unizar.es (R.M.-G.); 2 Cardiology Department, Hospital Universitario Miguel Servet, 50009 Zaragoza, Spain; jmcasadod@salud.aragon.es; 3 Universidad de Zaragoza, 50009 Zaragoza, Spain; jpuzo@unizar.es; 4 Endocrinology Department, Hospital Insular de Gran Canaria, 35016 Las Palmas de Gran Canaria, Spain; rosamariasanher@gmail.com; 5 Cardiology Department, Hospital Universitario Dr. Negrín, 35012 Las Palmas de Gran Canaria, Spain; martablanconuez@hotmail.com; 6 Lipid Clinic, Hospital Clinic, CIBEROBN, 08036 Barcelona, Spain; EORTEGA1@clinic.cat (E.O.-M.d.V.); MSITGES@clinic.cat (M.S.); 7 Lipid Unit, Hospital del Mar, 08003 Barcelona, Spain; JPedrobotet@parcdesalutmar.cat; 8 Lipid Unit, Hospital San Jorge, 22004 Huesca, Spain; teresabv11@hotmail.com; 9 Cardiology Department, Hospital San Jorge, 22004 Huesca, Spain

**Keywords:** aortic stenosis, aortic sclerosis, heterozygous familial hypercholesterolemia, statins, aortic valve calcification, LDL cholesterol

## Abstract

Hypercholesterolemia and statins are risk factors for aortic stenosis (AS) and vascular calcification, respectively. Whether heterozygous subjects with familial hypercholesterolemia (HeFH) treated with statins are at risk of AS is unknown. We study the prevalence of AS, aortic valve calcification (AoVC), and aortic sclerosis (ASc) in elderly subjects with HeFH in a prolonged statin treatment. Case-control study, cases were adults ≥65 years of age with a genetic diagnosis of HeFH, LDLc >220 mg/dl, and statin treatment ≥5 years. Controls were relatives of HeFH patients, with LDLc <190 mg/dl. Participants underwent a cardiac ultrasound for aortic valve analysis. We studied 205 subjects, 112 HeFH and 93 controls, with mean age 71.8(6.5) years and 70.0(7.3) years, respectively. HeHF, with respect to controls, presented greater gradients of aortic transvalvular pressure, 7.4(7.3) mmHg versus 5.0(2.8) mmHg, and maximum aortic velocity, 1.7(0.7) m/s versus 1.5(0.4) m/s, and lower aortic valve opening area, 2.0(0.7) cm^2^ versus 2.4(0.6) cm^2^ (all *p* < 0.05). AoVC and ASc were also more prevalent in HeFH (*p* < 0.05 between groups). Moderate/severe AS prevalence was higher among HeFH: 7.1% versus 1.1% (age- and sex-adjusted odds ratio (OR) 8.33, *p* = 0.03). Independent risk factors for aortic valve disease in HeFH were age and LDLc before treatment. The number of years under statin treatment was not associated with any aortic valve measurement. Subjects ≥65 years with HeFH in prolonged statin treatment show more aortic valvular disease and higher frequency of AS than controls. Life-long elevated LDLc exposure, rather than time of exposure to statins, explains this higher risk.

## 1. Introduction

Familial hypercholesterolemia is a common autosomal codominant disease mostly caused by mutations in the *LDLR* gene. The prevalence of heterozygous familial hypercholesterolemia (HeFH) is approximately of 1/200–500 in most countries [1]. Patients with HeFH show very high plasma concentration of low-density lipoprotein cholesterol (LDLc) [1,2,3] and, without lipid-lowering treatment, approximately 50% of HeFH men and 30% of HeFH women will develop coronary heart disease by the age of 50 years [1,4,5]. Fortunately, lipid-lowering treatment has decreased coronary heart disease in HeFH, and many patients now have almost a normal life expectancy [5]. Other diseases related to hypercholesterolemia, or to defects in the LDL receptor pathway, that are not reversed by statins or that may go unnoticed with shorter life-spans may appear nowadays thanks to the greater survival rate. This surge of new phenotypes has already been described in homozygous familial hypercholesterolemia. In the latter condition, these pediatric patients used to die from extremely premature coronary atherosclerosis. Given that the risk of coronary heart disease has been substantially reduced thanks to starting LDL apheresis from childhood [6,7,8], when homozygous familial hypercholesterolemia patients get older, they often show calcification of the aortic annulus and ascending aorta and an increased risk of severe aortic stenosis (AS) [7]. AS is an inflammatory and degenerative process caused by endothelial damage. The disease involves lipid infiltration, progressive fibrosis, and calcification, and ends up narrowing the aortic valve area [9]. Interestingly, AS prevalence has increased steadily in recent years in most countries, including Spain [10]. Risk factors for AS include age, hypercholesterolemia, diabetes mellitus, and hypertension, which are also traditional risk factors for arteriosclerosis [11]. Unfortunately, treatment for hypercholesterolemia with statins and ezetimibe has not proven to reduce AS progression in the long-term, although some benefit was observed only in a subset of patients with mild AS and high pretreatment LDLc levels [12,13]. Several factors may predispose HeFH patients to AS. They usually have very high LDLc concentration from childhood, a known risk factor for AS. In addition, chronic treatment with statins favors vascular calcification, which occurs when the lipidic and inflammatory components of the atheroma plaques are reduced by these drugs [14]. Besides, many HeFH patients have elevated lipoprotein(a) (Lp(a)) concentration, another well-known independent risk factor for vascular and aortic valve calcification (AoVC) [15,16]. Success of current treatment for HeFH may have allowed that these factors combine with the effects of aging. Nearly half of the elderly general population (>75 years old) have AoVC to some extent, and a fraction of them suffer AS [15]. Consequently, previous high early coronary heart disease (CHD) mortality in HeFH [17] could have hidden AoVC and AS that tend to appear at older ages. *LDLR* mutations strongly predict AoVC [18], but whether elderly HeFH patients are at higher risk of AS is still unknown. We hypothesized that many subjects with HeFH under chronic treatment with statins have not only AoVC, but also impaired hemodynamic parameters of the aortic valve function, even reaching AS diagnostic criteria. We aimed to study these functional differences by comparing HeFH patients with controls, to assess, in addition, the current prevalence of AS in HeFH subjects ≥65 years in chronic treatment with statins, and to explore potential risk factors for the development of AS in HeFH.

## 2. Methods

### 2.1. Study Characteristics

This is an observational, multicenter, case-control study. Five lipid clinics throughout Spain took part in the study. Consecutive HeFH cases were recruited with the following criteria: Age ≥65 years; a pathogenic mutation in a candidate gene for familial hypercholesterolemia (*LDLR*, *APOB,* or *PCSK9*) in the subject or in a first-degree relative; historic LDLc levels ≥220 mg/dl without lipid-lowering therapy; and statin treatment ≥5 years in all cases. Controls were selected from relatives of HeFH patients from the lipid clinics, requiring: Absence of hypercholesterolemia (LDLc <190 mg/dl without lipid-lowering treatment); age ≥55 years; and being either HeFH partners who cohabited >25 years or HeFH siblings. Additional HeFH cases were recruited from relatives of cases when they fulfilled the inclusion criteria. Participants were excluded if they had a personal history of rheumatic heart disease.

The key data collection component of the study was a transthoracic echocardiogram. Laboratory data were obtained from records in the lipid clinics from dates as close as possible (<1 year) to the cardiac ultrasound, if they were on stable lipid-lowering treatment. When these were not available, a blood sample was drawn during the visit. All procedures were carried out locally, at each lipid clinic.

All subjects gave a written informed consent to the protocol, which was approved by a central ethical committee (Comité Ético de Investigación Clínica de Aragón, CEICA).

### 2.2. Transthoracic Echocardiography

Transthoracic conventional echocardiograms were performed by cardiologists who were nationally certified for echocardiography. Echocardiogram studies were focused on the aortic valve at the same position for all patients. The following variables were measured: Mean aortic valve pressure gradient; maximum aortic velocity (Vmax); aortic valve area; aortic valve area indexed to body surface area; bicuspid or tricuspid aorta valve; valvular thickening >3 mm; and calcification of the aortic valve leaflets. The cardiologists performing echocardiograms were blinded to the diagnosis of HeFH. Degree of calcification of the aortic valve was scored as follows: 1, no calcification; 2, mildly calcified (small isolated spots); 3, moderately calcified (multiple larger spots); and 4, heavily calcified (extensive thickening and calcification of all cusps) [19]. AS was diagnosed as defined by the American College of Cardiology/American Heart Association Task Force on Practice Guidelines [11]. AS is considered present with any of the following findings: Mean aortic valve pressure gradient ≥20 mm; Vmax ≥2 m/s; and aortic valve area ≤1 cm^2^. Identified AS stages were mild (Vmax 2–2.9 m/s, valve pressure gradient <20 mm, and aortic valve area >1cm^2^); moderate (Vmax 3-3.9 m/s or valve pressure gradient 20–39 mm and aortic valve area >1 cm^2^), and severe (Vmax ≥4 m/s, valve pressure gradient ≥40 mm, or aortic valve area ≤1 cm^2^) [11]. Aortic valve sclerosis (ASc), a milder aortic condition, was defined in the presence of thickening (>3 mm) and/or calcification of the aortic valve without significant obstruction of flow (Vmax <2 m/s) or AS criteria [20]. 

### 2.3. Clinical Interview

Within clinical data information, we collected age, gender, level of education, history of smoking, hypertension, diabetes, personal history of cardiovascular disease and familial history of cardiovascular disease in first-degree relatives, age at which cardiovascular events occurred, lipid values without treatment, and history of lipid-lowering treatment. Level of education was classified as primary school, secondary school, and higher education. Current smoking was defined by smoking in the present or having smoked in the last year. Former smokers were defined as subjects having smoked at least 50 cigarettes in their lifetime, but not having smoked in the last year. Smoking burden was recorded as the number of daily packets smoked multiplied by the number of years smoked.

About the lipid-lowering treatment, we recorded age at which statin treatment began, statin most commonly prescribed, statin dose, ezetimibe use, age at which ezetimibe treatment began, and which statin and dose are prescribed as current treatment.

In participants with previous cardiovascular disease, age of first event and kind of event was recorded: Myocardial infarction; acute coronary syndrome requiring hospitalization; ischemic stroke; coronary, carotid, or peripheral revascularization; recovered sudden death; or aortic aneurysm.

### 2.4. Physical Exam

In the physical exam, we recorded height, weight, systolic and diastolic blood pressure, and presence of tendon xanthomas. Body mass index (BMI) was calculated as weight in kilograms divided by the square of height in meters.

### 2.5. Laboratory Tests

When current lipid values (<1 year) were not available, then a blood sample was obtained to determine cholesterol, triglycerides, HDLc, apolipoprotein B (apo B), Lp(a), glucose, and HbA1c. Laboratory measurements and sample preservation were performed at each center.

### 2.6. Definitions

Arterial hypertension was defined as systolic blood pressure ≥140 mm Hg, diastolic blood pressure ≥90 mm Hg, or current use of antihypertensive medication. Diabetes was defined as fasting plasma glucose ≥126 mg/dl, HbA1c ≥6.5%, or current use of antidiabetic medication.

### 2.7. Statistical Analysis

Summary data are expressed as mean (standard deviation) or percentage. For comparisons between cases and controls, aortic valve ultrasound variables and presence of aortic valve affectation levels are modeled in linear and logistic regressions based on generalized estimating equations (GEE) with exchangeable variance structure (to account for family links) with different levels of adjustment: Unadjusted, adjusted for sex and age, and additionally adjusted for untreated LDLc concentration. Analyses stratified by cases and controls and those restricted to cases were based on generalized linear models, and they included, in order to estimate their influence, the variable untreated LDLc concentration or years of life treated with lipid-lowering drugs. Influence of LDLc and lipid-lowering treatment were studied separately in cases and controls strata. All of the analyses were performed with the statistical software R version 3.4.4. and the package “gee” version 4.13.19.

## 3. Results

The research team recruited 205 subjects, 112 cases and 93 controls. Mean age was 71.8 years and 70.0 years in the case and control groups, respectively. Besides untreated total and LDLc, age, previous cardiovascular disease prevalence, and family history of premature cardiovascular disease prevalence were also higher in the HeFH group than in the control group. BMI was similar in both groups. There were no differences in smoking, hypertension, or type 2 diabetes mellitus (Table 1). All cases were on lipid-lowering treatment with a mean treatment time of 22.5 (8.7) years. No case or control had a history of aortic valve replacement.

### 3.1. Aortic Valve Characterization

**Figure 1:**
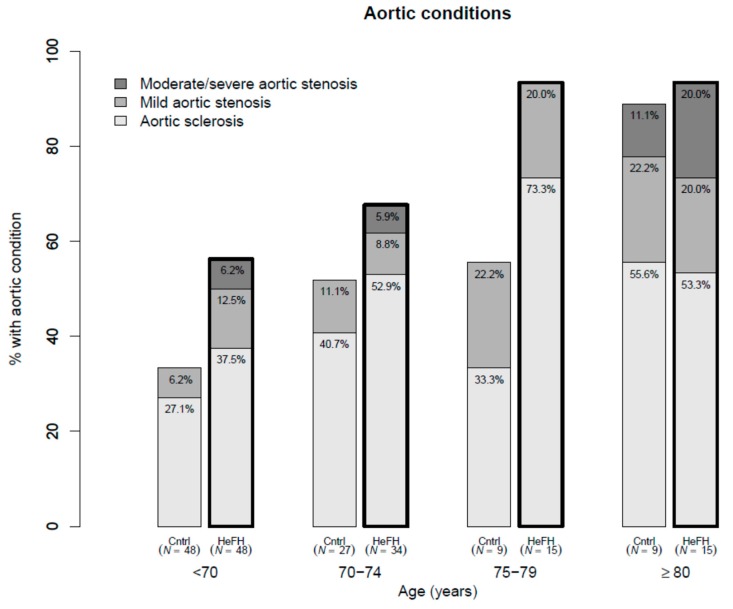
Prevalence of aortic stenosis and aortic sclerosis in heterozygous familial hypercholesterolemia (HeFH) and controls by age groups.

**Figure 2:**
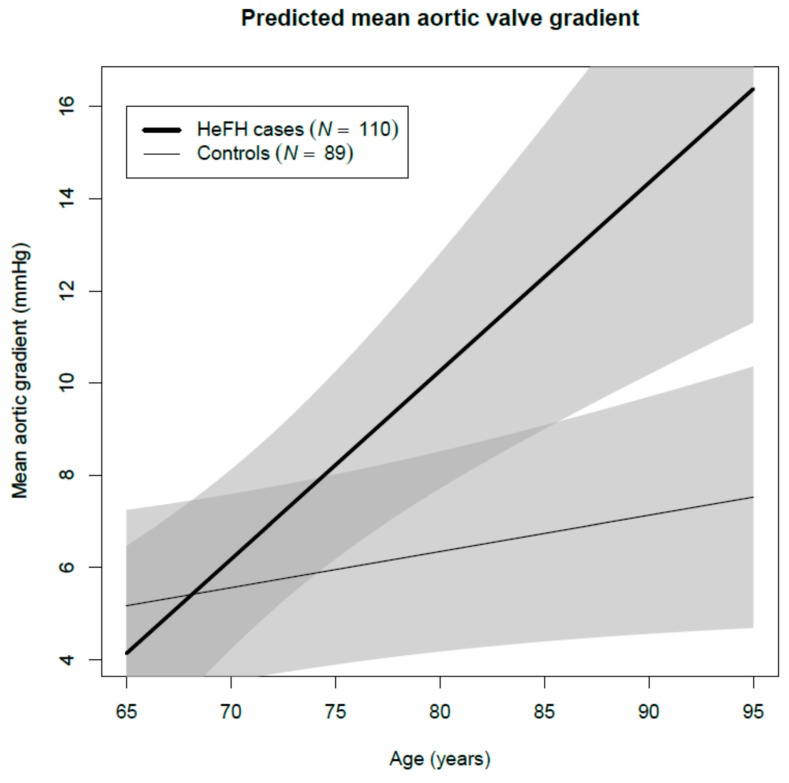
Predicted aortic valve gradient in HeFH and controls as a function of age. Models adjusted for sex and low-density lipoprotein cholesterol (LDLc) and stratified for the case and control groups.

Mean aortic valve pressure gradient was higher in cases (7.4 mmHg) than in controls (5.0 mmHg), after adjusting for age and sex (*p* = 0.002). HeFH patients, compared with controls, had greater maximum aortic velocity (Vmax) (1.7 m/s and 1.5 m/s, respectively, *p* = 0.011), and lower aortic valve area (2.0 cm^2^ and 2.4 cm^2^, respectively, *p* < 0.001). Among HeFH patients, average valvular calcification score of the aortic valve leaflets was higher and valvular thickening was more prevalent (*p* = 0.004). Left ventricular ejection fraction tended to be lower in cases (65.7% vs. 67.2%, *p* = 0.056). All studied valves were tricuspid. AS with moderate or severe criteria and ASc were more prevalent among HeFH (7% vs. 1%, age- and sex-adjusted OR 8.33, 95% confidence interval (CI) 1.22, 57.10, *p* = 0.031; and 55% vs. 32%, age- and sex-adjusted OR 1.90, 95% CI 1.04, 3.47, *p* = 0.061, respectively) (Table 2) and increased with age (Figure 1). Additionally, adjusting these comparisons of aortic measurements and stenosis prevalence for untreated LDLc concentrations rendered all differences non-significant. Thus, LDLc could justify a considerable amount of the valve differences between HeFH and controls, but LDLc is part of the definition of case and control, and stratified regressions were performed to clarify the issue. They showed that age, but not LDLc, was significantly associated with all aortic valve variables among controls, but Vmax and aortic valve calcification scores were also associated with untreated LDLc concentration among HeFH cases (Supplementary Table S1). Mean aortic valve gradient increased 4.1 (2.1, 6.1) mmHg/10 year among cases, while it only increased 0.8 (0.0, 1.6) mmHg/10 year among controls across different age groups (Supplementary Table S1 and Figure 2). All clinical and laboratory data were similar in all aortic valve stages except for the presence of tendon xanthomas (Supplementary Table S2).

### 3.2. Risk Factors for Valvular Disease

In order to evaluate how statin treatment could modify aortic valve parameters in HeFH patients, we used models including sex, age, and length of statin treatment among HeFH patients. Aortic valve area decreased, and aortic valve calcification score increased significantly with age (*p* < 0.001), independently of statins (Table 3). Ejection fraction was independent of age but decreased with length of statin treatment (*p* = 0.005). Mean aortic valve gradient increased with age in cases and controls, but with higher incremental rate in HeFH (Figure 2).

## 4. Discussion

In the present study, we described the prevalence of aortic disease in HeFH patients ≥65 years under long-term treatment with lipid-lowering drugs. Aortic valve involvement in HeFH has been previously explored, but, to our knowledge, this is the first work focused on elderly subjects, the most clinically relevant population due to the close relationship of AS with age, and the first to describe the prevalence of AS and to evaluate the potential role of statin treatment in development of aortic disease in HeFH. Our results are in agreement with those recently published from a registry-based prospective cohort study in Norway, in which a marked increased risk of AS in HeFH was found compared with the general Norwegian population [21].

### 4.1. Prevalence of AS in HeFH

In our study, AS prevalence is more than three times higher than that reported for this group of age in the general population, 1.5–3%, and also higher than in our related control sample [22,23,24,25]. In view of this substantially elevated prevalence, a systematic transthoracic echocardiography is probably justified for the screening of AS in elderly HeFH over 65 years.

Several studies have previously analyzed aortic valve thickening or calcification in HeFH. Ten Kake et al. compared a cohort of 59 HeFH subjects from the Netherlands with controls and showed that the patients with HeFH, especially those with *LDLR*-negative mutations, showed higher AoVC prevalence (41% versus 21%, respectively, *p* < 0.001) [18]. This ratio is similar to the one observed in our study. However, the authors did not report data on AS, probably because their sample was smaller than ours and it included younger patients. In the Cardiovascular Health Study, clinically defined familial hypercholesterolemia was associated with AoVC and ASc, but an association with AS could not be demonstrated [26,27]. In our study, in addition to fulfilling clinical lipidic criteria, all cases were defined by having a genetic mutation in a gene that causes familial hypercholesterolemia, ascertained directly in them or in a blood-relative. It is well established that familial hypercholesterolemia definition based exclusively on clinical criteria includes other forms of genetic hypercholesteremia [28,29], and hypercholesterolemic subjects with a genetic mutation have more severe cardiovascular phenotypes than in the absence of mutation, even with a similar LDLc concentration at the moment of diagnosis [30].

### 4.2. Risk Factors for Aortic Valvular Disease

Our results support that atherosclerosis risks factors, including high LDLc throughout life, are major risk factors for AS [31]. AS is probably produced by a combination of mechanical stress and endothelial damage of the aortic valves, driving to subsequent valve inflammation, fibrosis, calcification, and progressive valve narrowing [9]. Mechanical stress affects the endothelial function and facilitates infiltration of lipids and inflammatory cells (T-cells) into the valve. All of these mechanisms are involved in the inflammatory activity [32,33]. As a result, fibroblasts differentiate into myofibroblasts, which, under the influence of angiotensin, promote thickening of the valve [9]. Hypercholesterolemia treatment does not prevent progression from moderate to severe AS once AS is already established [34], suggesting that hypercholesterolemia plays a role in the startup process but has little effect in the advanced disease [35]. Our study would support current guidelines, which recommend early and intensive treatment of HeFH to prevent not only coronary disease, but also valvular disease later in life [5].

### 4.3. Statins and Valvular Disease

Lipid-lowering treatment is associated with increased vascular calcification [36] due, at least in part, to statins-enhanced new bone formation in bone cells, via increased expression of gene *BMP*-2 [37]. Furthermore, the reduction in inflammatory markers associated with statins is significantly associated with the percentage of calcium volume within the coronary plaques [38]. Consequently, it has been speculated that lipid-lowering treatment, especially prolonged statin treatment, may favor development of AoVC and AS [39]. Although, other studies such as Al Kindi’s showed that vascular calcification associated to statins is not related to valve calcification [40]. Currently, HeFH are the patients to whom earlier, more intense, and more prolonged lipid-lowering drugs are prescribed. Hence, our elderly HeFH sample, exposed to statins for a mean of 22.5 years, is excellent to study this potential association. We found that the number of years under statin treatment is not a risk factor for AS, indicating that if there is any risk of AS with statin, it is highly compensated by the beneficial effect on LDLc, as it has been previously suggested [22]. These results are in full agreement with those found in the Simvastatin and Ezetimibe in Aortic Stenosis (SEAS) study, where, in a non-prespecified post-hoc analysis, lipid-lowering treatment impeded the progression of AS in patients with the highest LDL cholesterol concentration ( >160 mg/dL) and mild AS at baseline [41]. Statin therapy was also significantly associated with a lesser change in aortic valve area in SALTIRE and RAAVE trials [42].

### 4.4. Study Limitations

This was a multicenter project in which echocardiographic studies were performed by different researchers, and thus a certain degree of variability may exist in the measurements. However, all ultrasound scans were performed by cardiologists who were experts in echocardiography, with practice within hospitals of the Spanish National Health System, and all of them certified at the national level with homogeneous and strict criteria. In addition, echocardiographers were blinded to the diagnosis of HeFH to avoid bias.

Measurements of aortic valve functionality may vary in the presence of abnormal systolic function [43]. Although it was not an exclusion criterion, none of our cases and controls had left ventricular ejection fraction (LVEF) <40% or hypertrophic cardiomyopathy, thus, this limitation does not play an important role in our study, and all measurements of the valvular surface could be performed by continuity equation, the best standardized procedure. Finally, some data on the personal history of lipid-lowering drug use and family history of cardiovascular disease were self-reported by the participants, and they may have been affected by some degree of recall inaccuracy. However, the main outcome and the inclusion and exclusion criteria for the cases and controls groups were based on objective data, collected or verified by the research team.

It would had been important to have the lifetime LDLc exposure to study the effect of cumulative LDLc on AS. Considering that we are dealing with elderly patients, this is not feasible. However, we considered LDLc at diagnosis and the number of years under statin treatment, and we believe that both variables are good subrogates of lifetime LDLc exposure.

## 5. Conclusions.

Subjects ≥65 years with HeFH in prolonged statin treatment for a mean period of over 22 years show more aortic valvular disease and higher frequency of AS than controls. Independent risk factors for aortic valve disease in HeFH were age and LDLc before treatment. Duration of statin treatment was not associated with any aortic valve measurement. Hence, cumulative LDLc exposure, rather than time of exposure to statins, explains this higher risk. These results suggest that elderly HeFH should be monitored for the presence of aortic disease, and emphasize the importance of early lipid-lowering treatment in HeFH population to prevent not only coronary disease, but also aortic valvular disease. Furthermore, our study provides additional support to the already established body of work on the role of hypercholesterolemia on AS.

## Figures and Tables

**Table 1:** Baseline clinical and laboratory characteristics of cases and controls.

Mean (SD) / proportion (*n* = count)		Controls		Cases	
	*n*	Mean/%	*n*	Mean/%	*p*-value
Age (years)	93	70.0 (7.3)	112	71.8 (6.5)	0.038
Sex, women % (*n* = count)	93	51.6 (*n* = 48)	112	66.1 (*n* = 74)	0.050
Weight (Kg)	92	75.9 (14.3)	110	72.1 (13.5)	0.054
Height (cm)	92	161.7 (8.5)	110	159.4 (9.9)	0.075
Systolic blood pressure (mmHg)	92	136.7 (14.9)	110	134.1 (16.9)	0.199
Diastolic blood pressure (mmHg)	92	79.5 (9.7)	110	77.1 (9.7)	0.089
Body mass index (Kg/m^2^)	92	29.0 (4.7)	110	28.3 (4.0)	0.265
Tendon xanthomas, % (*n* = count)	93	0.0 (*n* = 0)	102	39.2 (*n* = 40)	<0.001
Hypertension, % (*n* = count)	93	55.9 (*n* = 52)	112	54.5 (*n* = 61)	0.769
Type 2 diabetes, % (*n* = count)	93	14.0 (*n* = 13)	112	21.4 (*n* = 24)	0.139
Previous cardiovascular disease, % (*n* = count)	93	16.1 (*n* = 15)	112	27.7 (*n* = 31)	0.036
Family history of premature cardiovascular disease, % (*n* = count)	89	25.8 (*n* = 23)	100	45.0 (*n* = 45)	<0.001
Packages/day × number of years smoking	90	15.2 (25.3)	108	9.8 (21.6)	0.120
Lipid-lowering treatment, % (*n* = count)	93	51.6 (*n* = 48)	112	100.0 (*n* = 112)	<0.001
Statin treatment (years)	48	8.3 (7.2)	110	22.5 (8.7)	<0.001
Ezetimibe treatment, % (*n* = count)	68	8.8 (*n* = 6)	112	83.0 (*n* = 93)	<0.001
Untreated total cholesterol (mg/dL)	93	223.8 (43.3)	111	395.9 (73.0)	<0.001
Untreated triglycerides (mg/dL)	93	145.7 (119.0)	112	139.7 (76.3)	0.669
Untreated HDLc (mg/dL)	91	56.9 (15.3)	112	55.7 (13.6)	0.621
Untreated LDLc (mg/dL)	90	138.0 (31.7)	111	314.2 (71.4)	<0.001

Continuous data are expressed as mean (standard deviation, SD); categorical data are expressed as percentages (*n* = count). HDLc: High-density lipoprotein cholesterol; LDLc: Low-density lipoprotein cholesterol. *p*-values from linear and logistic regressions based on generalized estimating equations (GEE) with exchangeable variance structure, unadjusted.

**Table 2:** Morphological and hemodynamic parameters of aortic valve in cases and controls.

Mean (SD)/proportion (*n* = count)	Controls		Cases				
	*n*	mean/%	*n*	mean/%	*p*-value *	*p*-value **	*p*-value ***
Mean aortic valve pressure gradient (mm)	92	5.0 (2.8)	111	7.4 (7.3)	0.002	0.003	0.626
Maximum aortic velocity (Vmax) (m/s)	92	1.5 (0.4)	111	1.7 (0.7)	0.004	0.011	0.959
Aortic valve area (cm^2^)	92	2.4 (0.6)	107	2.0 (0.7)	<0.001	<0.001	0.771
Left ventricular ejection fraction (%)	93	67.2 (7.0)	111	65.7 (9.5)	0.167	0.056	0.285
Aortic valve velocity ratio	61	0.73 (0.11)	61	0.70 (0.14)	0.244	0.138	0.321
Calcification of the aortic valve leaflets (score)	92	0.7 (0.9)	110	1.1 (1.0)	0.002	0.008	0.723
Valvular thickening >3 mm, % (*n* = count)	93	12.9 (*n* = 12)	112	27.7 (*n* = 31)	0.006	0.004	0.600
Aortic stenosis, % (*n* = count)	93	11.8 (*n* = 11)	112	20.5 (*n* = 23)	0.107	0.171	0.842
Aortic stenosis moderate or severe, % (*n* = count)	93	1.1 (*n* = 1)	112	7.1 (*n* = 8)	0.067	0.031	0.187
Aortic sclerosis, % (*n* = count)	92	34.8 (*n* = 32)	111	49.5 (*n* = 55)	0.046	0.061	0.337

Continuous data are expressed as mean (SD); categorical data are expressed as percentages (*n* = count). *p*-values from linear and logistic regressions based on generalized estimating equations (GEE) with exchangeable variance structure. *p*- value *: Unadjusted; *p* -value **: Adjusted for sex and age; *p*-value ***: Adjusted for sex, age, and untreated LDLc concentration.

**Table 3:** Influence of age and length of lipid-lowering treatment on aortic valve characteristics in HeFH.

Morphological and hemodynamic parameters	Cases			
	Per each 10 year of age Difference/OR (95% CI)	*p*-value	Per each 10 year of lipid-lowering use Difference/OR (95% CI)	*p*-value
Mean aortic valve pressure gradient (mm)	4.623 (2.518, 6.728)	<0.001	0.214 (−1.304, 1.732)	0.783
Maximum aortic velocity (Vmax) (m/s)	0.394 (0.185, 0.603)	<0.001	0.012 (−0.139, 0.162)	0.881
Aortic valve area (cm^2^)	−0.373 (−0.562, −0.185)	<0.001	0.001 (−0.133, 0.135)	0.990
Left ventricular ejection fraction (%)	−1.924 (−4.634, 0.787)	0.167	−2.877 (−4.827, −0.927)	0.005
Calcification of the aortic valve leaflets (score)	0.698 (0.418, 0.979)	<0.001	−0.034 (−0.233, 0.165)	0.737
Valvular thickening >3 mm, OR	1.36 (0.69, 2.63)	0.359	0.86 (0.52, 1.41)	0.549
Aortic stenosis, OR	2.11 (1.04, 4.37)	0.038	0.94 (0.52, 1.65)	0.817
Aortic stenosis moderate or severe, OR	2.95 (1.07, 8.26)	0.032	0.82 (0.29, 2.15)	0.687
Aortic sclerosis, OR	1.23 (0.67, 2.32)	0.510	1.03 (0.66, 1.62)	0.883

Linear and logistic regressions based on generalized linear models (GLM). HeFH: heterozygous familial hypercholesterolemia; OR: odds ratio; CI: Confidence Interval. *p-*value from a single model, adjusted for sex.

## Supplementary Materials

The following are available online at https://www.mdpi.com/2077-0383/8/12/2209/s1, Table S1: Influence of age and low-density lipoprotein cholesterol (LDLc) on morphological and hemodynamic parameters of aortic valve in cases and controls, Table S2: Baseline clinical and laboratory characteristics of HeFH with aortic valve conditions.

## Abbreviations

AoVC	Aortic valve calcification
AS	Aortic stenosis
ASc	Aortic sclerosis
BMI	Body mass index
GEE	Generalized estimating equations
HeFH	Heterozygous familial hypercholesterolemia
HDLc	High-density lipoprotein cholesterol
LDLc	Low-density lipoprotein cholesterol
Lp(a)	Lipoprotein(a)
Vmax	Maximum aortic velocity
