# Supplementary material for: Aortic Valvular Disease in Elderly Subjects with Heterozygous Familial Hypercholesterolemia: Impact of Lipid-Lowering Therapy
Source: J Clin Med. 2019 Dec 14;8(12):2209. doi: 10.3390/jcm8122209 (PMC6947617; doi:10.3390/jcm8122209)
Supplement: Supplementary file 1 [file jcm-08-02209-s001.pdf]

Supplementary table 1. Influence of age and LDLc on morphological and hemodynamic parameters of aortic valve in cases and controls.

**Morphological and hemodynamic parameters**	**Controls**				**Cases**			
	**Per each 10 year Diff/OR (95% CI)**	**P-value**	**Per each 10 mg/dl of cLDL Diff/OR (95% CI)**	**P - value**	**Per each 10 year Diff/OR (95% CI)**	**P - value**	**Per each 10 mg/dl cLDL Diff/OR (95% CI)**	**P -value**
Mean aortic valve pressure gradient (mm)	0.785 (-0.023, 1.592)	0.060	0.067 (-0.138, 0.272)	0.525	4.085 (2.077, 6.093)	<0.001	0.087 (-0.096, 0.269)	0.353
Maximum aortic velocity (Vmax) (m/s)	0.120 (0.003, 0.237)	0.048	0.014 (-0.016, 0.044)	0.365	0.341 (-0.143, 0.539)	0.001	0.012 (0.006, 0.030)	0.187
Aortic valve area (cm2)	-0.257 (-0.396, -0.117)	0.001	-0.024 (-0.059, 0.012)	0.189	-0.328 (-0.506, -0.150)	<0.001	-0.013(-0.029, 0.002)	0.099
Left ventricular ejection fraction (%)	2.569 (0.597, 4.541)	0.012	0.072 (-0.408, 0.552)	0.770	-2.515 (-5.203, 0.173)	0.069	0.012 (-0.231, 0.255)	0.923
Calcification of the aortic valve leaflets (score)	0.489 (0.249, 0.729)	<0.001	0.033 (-0.028, 0.094)	0.288	0.682(0.422, 0.941)	<0.001	0.026 (0.003, 0.049)	0.028
Valvular thickening >3 mm, % [n]	3.26 (1.31, 8.81)	0.013	1.18 (0.94, 1.54)	0.182	1.26 (0.66, 2.36)	0.463	1.03 (0.97, 1.09)	0.304
Aortic stenosis, % [n]	2.63 (1.13, 6.48)	0.027	0.97 (0.79, 1.22)	0.817	1.93 (0.98, 3.81)	0.054	1.04 (0.98, 1.11)	0.190
Aortic stenosis moderate or severe, % [n]	-		-		2.65 (1.01, 6.90)	0.041	1.02 (0.91, 1.13)	0.684
Aortic sclerosis, % [n]	1.95 (1.02, 3.92)	0.049	1.02 (0.87, 1.20)	0.797	1.30 (0.72, 2.40)	0.391	1.01 (0.96, 1.07)	0.691

Linear and logistic regressions based on generalized linear models (GLM) stratified by cases and controls. CI: Confidence Interval. P-value: adjusting for age and sex

Supplementary table 2. Baseline clinical and laboratory characteristics of HeFH with aortic valve conditions

**Mean (SD) / proportion [n]**		**Normal valve**		**Aortic sclerosis**		**Aortic (mild)**		**Aortic stenosis (moderate or severe)**	
	**N**	**mean/%**	**N**	**mean/%**	**N**	**mean/%**	**N**	**mean/%**	**P -value**
Age (years)	34	69.2 (3.9)	55	72.4 (6.3)	15	72.8 (6.5)	8	76.1 (12.0)	0.002
Sex, women % [n]	34	61.8 [21]	55	70.9 [39]	15	66.7 [10]	8	50 [4]	0.574
Systolic blood pressure (mmHg)	34	131.8 (15.2)	54	133.9 (16.2)	15	135.8 (19.2)	7	143.3 (25.4)	0.044
Diastolic blood pressure (mmHg)	34	77.7 (9.1)	54	76.6 (10.5)	15	78.5 (9.6)	7	74.6 (8.3)	0.993
Body mass index (Kg/m2)	34	27.9 (3.5)	53	28.1 (4.2)	15	30.1 (4.7)	8	27.1 (2.9)	0.811
Tendon xanthomas, % [n]	30	26.7 [8]	50	42.0 [21]	14	42.9 [6]	8	62.5 [5]	0.027
Hypertension, % [n]	34	38.2 [13]	55	54.5 [30]	15	86.7 [13]	8	62.5 [5]	0.024
Type 2 Diabetes, % [n]	34	11.8 [4]	55	23.6 [13]	15	26.7 [4]	8	37.5 [3]	0.093
Previous cardiovascular disease, % [n]	34	17.6 [6]	55	30.9 [17]	15	33.3 [5]	8	37.5 [3]	0.300
Family history of premature cardiovascular disease, % [n]	31	32.3 [10]	52	46.2 [24]	13	69.2 [9]	4	50 2]	0.036
Packages/day · years	33	10.3 (17.9)	53	8.4 (20.6)	15	11.8 (32.4)	7	12.7 (21.1)	0.880
Statin treatment (years)	34	22.4 (8.6)	53	22.9 (9.3)	15	22.5 (8.9)	8	20.9 (5.7)	0.906
Ezetimibe treatment, % [n]	34	79.4 [27]	55	81.8 [45]	15	86.7 [13]	8	100 [8]	0.281
Untreated total cholesterol (mg/dl)	33	380.7 (58.4)	55	398.7 (79.3)	15	416.4 (77.8)	8	401 (74.2)	0.119
Untreated triglycerides (mg/dl)	34	143.1 (78.4)	55	139.5 (81.9)	15	134.8 (62.4)	8	136.4 (60.2)	0.675
Untreated HDLc (mg/dl)	34	55.8 (13.9)	55	56.3 (13.9)	15	54.2 (12.9)	8	54.4 (13.3)	0.787
Untreated LDLc (mg/dl)	34	296.7 (59.6)	54	318.3 (76.5)	15	335.2 (76.7)	8	321.4 (68.6)	0.091
Lp(a) (mg/dl)	18	51.8 (54.1)	26	67.7 (71.6)	9	45.6 (46.1)	6	55.1 (45.4)	0.888

Continuous data presented as mean ± SD; categorical data presented as percentages. BP: blood pressure, BMI: body mass index, HDLc: high-density lipoprotein cholesterol, LDLc: low-density lipoprotein cholesterol. P -value from linear and logistic regressions based on generalized linear models (GLM), adjusted for age and sex
